# Visual ERP-based brain–computer interface use with severe physical, speech and eye movement impairments: case studies

**DOI:** 10.1186/s12984-025-01836-0

**Published:** 2025-12-21

**Authors:** Arne Van Den Kerchove, Juliette Meunier, Marie de Moura, Alixe Willemssens, Dorien Geeurickx, Edward Schiettecatte, Philip Van Damme, Hakim Si-Mohammed, François Cabestaing, Etienne Allart, Marc M. Van Hulle

**Affiliations:** 1 Laboratory for Neuro-and Psychophysiology, Department of Neurosciences, Leuven Brain Institute, Leuven.AI, KU Leuven, Campus Gasthuisberg, Herestraat 49 bus 1021, 3000 Leuven, Belgium; 2 CNRS, Centrale Lille, UMR 9189 CRIStAL, Bâtiment ESPRIT, University of Lille, Avenue Henri Poincaré, 59655 Villeneuve d’Ascq, France; 3 Fondation Partage et Vie, 24 Rue des Fleurs, 59120 Loos, France; 4 TRAINM Clinics, Quellinstraat 38, 2018 Antwerp, Belgium; 5 Department of Neurology, University Hospitals Leuven, Campus Gasthuisberg, Herestraat 49, 3000 Leuven, Belgium; 6 CHU de Lille, Service de Rééducation Neurologique Cérébrolésion, UFR3S médecine, Lille Neuroscience and Cognition, Hôpital Swynghedauw, University of Lille, Rue André Verhaeghe, 59000 Lille, France

**Keywords:** Brain–computer interface, Augmentative and alternative communication (AAC), Event-related potentials, Covert visuospatial attention, Eye motor impairment

## Abstract

**Background:**

Individuals who experience severe speech and physical impairment face significant challenges in communication and daily interaction. Visual brain–computer interfaces (BCIs) offer a potential assistive solution, but their usability is limited when facing restrictions in eye motor control, gaze redirection and fixation. This study investigates the feasibility of a gaze-independent visual oddball BCI for use as an augmentative and alternative communication (AAC) device in the presence of limited eye motor control.

**Methods:**

Seven participants with varying degrees of eye motor control were recruited and their conditions were thoroughly assessed. Visual oddball BCI decoding accuracy was evaluated with multiple decoders in three visuospatial attention (VSA) conditions: overt VSA, with fixation cued on the target, covert VSA, with fixation cued on the center of the screen, and free VSA without gaze cue.

**Results:**

covert VSA with central fixation leads to decreased accuracy, whereas free VSA is comparable to overt VSA for some participants. Furthermore, cross-condition decoder training and evaluation suggests that training with overt VSA may improve performance in BCI users experiencing gaze control difficulties.

**Conclusions:**

These findings highlight the need for adaptive decoding strategies and further validation in applied settings in the presence of gaze impairment.

## Background

Neurological conditions, such as acquired brain lesions, neuromuscular diseases and Amyotrophic Lateral Sclerosis (ALS) can result in speech and physical impairment (SPI). This, in turn, significantly alters their participation in daily activities and communication, sometimes affecting their quality of life and self-reliance. Augmentative and alternative communication (AAC) technology [1–3] leveraging visual brain–computer interfaces (BCIs) [4, 5], which relies on the interpretation of visual stimuli by the user, offers several advantages in this context. The rapid stimulation pace of visual BCIs and the modulation of information over spatial attention allow for high information transfer rates (ITRs) [6, 7]. Combined with their ability to operate with non-invasive recording technology, this makes them well-suited for communication tasks.

However, visual and eye motor differences such as nystagmus (uncontrolled eye movements), diplopia (double vision), ophthalmoplegia (partial or complete eye paralysis), fatigability and a limited range of head motion can significantly interfere with accurate control of eye gaze, reducing performance with BCIs that rely on visual cues [8–11]. Unfortunately, it is again for this group that eye tracking solutions also perform poorly, making them more reliant on potential developments in BCIs that do not rely on eye gaze.

Hence, some BCI and AAC solutions rely on the capability of the user to direct their gaze at a desired target. In this case, the gaze fixation of the user and their visuospatial attention (VSA) coincide (overt VSA). With the development of BCI-AAC for users with eye movement impairment in mind, alternative solutions can circumvent gaze redirection by designing gaze-independent BCIs [12], which either avoid visual stimulation or exploit some form of covert VSA, where gaze and VSA do not coincide [13–17].

Several studies on visual oddball BCIs indicate that performance declines when relying on covert VSA [18–20]. The tasks performed in these studies emulate covert VSA using central gaze fixation. This implicitly assumes that BCI users with eye motor impairment who would benefit from gaze-independence, would feel comfortable performing this central fixation. In other words, one could argue that a BCI verified to work only with central fixation, could also be considered gaze-dependent. It does not account for the remaining eye motor capabilities of individuals experiencing SPI and most forms of eye movement difficulties, the (dis)comfort experienced when fixating, among other challenges and confounding factors related to eye motility. The aforementioned works improved gaze-independent BCIs in various ways or discovered obstacles preventing accurate gaze-independent BCI use. However, it is notable that most studies reporting on gaze-independent visual BCIs were performed with participants without neuromuscular conditions, and only very few report on BCI use of participants with speech, physical and gaze impairment (SPGI). Results are usually different from those obtained with control participants without neuromuscular conditions, due to differences in capabilities, brain response, experimental equipment and environment.

Combaz et al. [21] showed that steady-state visually evoked potential (SSVEP) and oddball paradigm BCIs can be used effectively by individuals living with Locked-in Syndrome (LiS). Lesenfants et al. [22] tested a BCI using gaze-independent SSVEPs in six participants with LiS, yet only two of them exceeded chance level accuracy. More recently, Peters et al. [23] performed a trial with two participants living with a more progressed stage of ALS with visual impairment. Their SSVEP paradigm was not optimized for gaze-independence, but the system showed high accuracy, outperforming an eye tracking alternative. It would be of interest to determine whether such results can be replicated with participants with other neurological conditions, and with a visual oddball rather than an SSVEP paradigm.

Orhan et al. [24] and Oken et al. [25] tested the rapid serial visual presentation (RSVP) speller with participants with LiS. Due to the serial nature of the stimulation paradigm, communication was rather slow but they demonstrated the feasibility of the RSVP paradigm in a relevant target population.

Severens et al. [26] evaluated the visual Hex-o-Spell [19] on 5 participants living with ALS and showed that this visual oddball interface optimized for gaze-independence can outperform a tactile BCI. While this speaks to the power of visual paradigms, even in groups that are expected to have eye motor impairment, they did not verify the gaze direction of participants during the experiment. It was assumed that participants were performing overtly. Participants living with ALS also exhibited a substantially lower accuracy than healthy controls (58% vs. 88%).

Previously mentioned approaches focus mainly on optimizing the stimulation paradigm and visual layout. However, an alternative approach was proposed by Aloise et al. [27]. They retained classical paradigms with proven effectiveness but adapted decoding strategies to enhance decoding performance of covert VSA. While the attempts of Aloise et al. [27] did not report any classifiers improving covert decoding, Aricò et al. [28] propose a method relying on properties of the covert VSA event-related potential (ERP). Aricò et al. [28] identified that latency jitter in the P3 ERP component is significantly higher when performing covert VSA compared to overt VSA for multiple ERP stimulation paradigms. Hence, they proposed an adapted decoding scheme which aligns the relevant ERP components over trials. While they show the potential effectiveness of this approach, they stopped short of building an applicable decoder. Hardiansyah et al. [29] also leveraged the distribution of ERP latencies to decode covert VSA.

Van Den Kerchove et al. [30] built on this latency jitter property to implement an applicable decoder to improve gaze-independent decoding for the visual Hex-o-Spell interface. They propose a classifier using temporal alignment to improve decoding performance in the presence of jitter. Furthermore, this study also partially accounted for the fact that BCI users with SPGI might not fully rely on central gaze fixation by evaluating settings independent of central fixation. They showed that gaze-independent performance can be improved in healthy subjects by using a suited decoding strategy that corrects for latency jitter in covert VSA responses. Yet, there is a need for verification of these results in the presence of SPGI.

Ultimately, this line of research aims to further the development of gaze-independent BCI for individuals with progressed stages of LiS. These users have generally no practical means of communication other than BCIs. Furthermore, the recruitment of potential users can be challenging, since they are scarce and may have extensive accessibility needs and may experience challenges in performing the experiment task when participating in a study [31]. We also notice that existing BCIs solutions often follow a strict dichotomy between overt and covert VSA with cued fixation. It is not obvious that such cases occur in natural visual BCI operation by individuals with SPGI. Individuals experiencing only partial loss of voluntary eye motor control or who live with a less progressed stage of a neuromuscular disease can still control their gaze to some extent, but experience difficulties operating classical AAC technology based on eye tracking. Hence, there is a need to verify if earlier (BCI) AAC methods and concepts actually apply for them.

Based on literature and our experience, we formulate the following research questions. (1) Do BCI users with SPGI naturally rely on covert or overt VSA, or a mix of both, (2) How well do they perform using a visual BCI? (3) Do they also benefit from decoding approaches improving covert VSA decoding? (4) Can effective calibration techniques be crafted by relying on residual eye motor control? To this end, we apply the concepts from earlier work and literature, especially the WCBLE decoder and Hex-o-Spell visual oddball interface, in the presence of SPI and various differences in eye motor control. The contributions of this study are as follows: (1) the recruitment of individuals that specifically have SPGI in a visual BCI study and the exploration of their eye motility and experienced comfort when operating such a BCI, (2) a baseline evaluation of the decoding performance for this group, (3) the verification of the gaze-independent decoding strategy proposed by Van Den Kerchove et al. [30], (4) and a calibration strategy suitable for these users. Finally, we assess the clinical relevance and formulate a set of recommendations based on our experiences when carrying out this study. These insights could be of interest when designing further similar studies or BCI-AAC solutions for this target user population.

## Methods

### Recruitment and study design

Participants were recruited from the Neuromuscular Reference center at University Hospitals Leuven (Leuven, Belgium), TRAINM Clinics (Antwerp, Belgium), the Neurorehabilitation Unit at the Lille University Medical Center (Lille, France), and Fondation Partage et Vie (Loos, France). Experiments were performed after prior informed consent and under the supervision of the treating physician, with a protocol prior approved by the Ethics Commission of the University Hospitals Leuven (S62547).

We partially base the recruitment criteria on the BCI user classification system presented by Wolpaw et al. [31]. They present a three-class system: (1) complete LiS without voluntary movements (including eye movements), (2) the ability to control a single-button switch using very limited voluntary movement, consisting of eye movements or small limb movements, and (3) reduced but substantial voluntary movements (either speech or limb control), able to operate a range of classical AAC devices.

In order to be included in the study, participants must:

1. be at least 18 years old and no older than 60 years,
2. belong to class 2 or 3 according to the aforementioned BCI user classes,
3. have limitations to the extent or comfort of their eye motor control (partial or full gaze paralysis, uncontrolled gaze movements, or conditions of the capability to direct the gaze or fixate),
4. have given their informed consent prior to participation.

Participants were excluded if:

1. they had any diagnosis of a major medical condition other than neuromuscular conditions covered by inclusion criteria 2 and 3,
2. they had a predisposition to or a history of any kind of epileptic seizures, including photosensitive epilepsy,
3. they had a severe loss in vision or hearing that would significantly impair participation in the experiment,
4. they were using specific psychoactive medications or substances that could affect the outcome (neuroleptics or benzodiazepines),
5. the instructions or the experimental tasks could not be made accessible for participation
6. they experienced any other limitations preventing them from performing the given task.

The study was conducted in two visits. The first visit served to verify the inclusion and exclusion criteria, to explain the context and course of the study, and answer the participant’s questions in cooperation with the paramedical team. Afterwards, the informed consent form was signed. Initial clinical and eye motor assessment was also performed in the first visit. The first visit was conducted by the clinician or paramedical team or by the experimenting researchers. If a participant was included, the second visit consisted of eye motor and visual assessment, the electroencephalography (EEG) setup, eye tracker setup and BCI stimulation and recording. The second visit lasted around two to three hours.

Clinical assessment was based on a questionnaire polling for basic demographic and neuromuscular questions, answered by the participant or the paramedical team. This questionnaire is provided in  appendix B. Other relevant details concerning communication, pragmatics and presentation of the patient were observed by the researchers who carried out the experiments or through conversation with the participant or the paramedical team. The clinical assessment was complemented with further relevant details provided by the supervising clinician.

We also asked the participants to evaluate their mood and tiredness on a scale from 1 to 10 before and after the experiment conducted in the second session. This should serve as a preliminary indication of comfort and task load, since it was impractical to conduct more extensive surveys.

### Vision and eye motor examination

Eye motor and visual differences assessments were obtained through self-reporting using the questions presented in appendix C and completed with details from the medical file or from an oculomotor assessment provided by the clinician where possible. Vision and eye motor control were assessed using the ‘visual skills’ framework established by Fried-Oken et al. [9]. These describe on a higher level the factors that can reduce visual BCI performance when impaired. Visual acuity was assessed using a logMAR chart [32]. During this assessment as well as during the rest of the experiment session, participants wore their corrective glasses or lenses if they used any. In Table 2, the distinction between minor and major impairment was made according to the following rule: Major impairment implies the impairment was complete, or the given ability on its own prevents or nearly prevents the participant from operating a regular visual oddball BCI as a healthy control subject would, after potential visual corrections. Minor impairment implies the impairment is limited to discomfort or does not on its own strictly prevent the user from operating a visual BCI as a healthy control subject would, after potential corrections. Finally, we also recorded gaze position throughout the experimental session to register the participant’s gaze relative to the stimulated BCI targets, as described below in Sect. 2.4.

### BCI stimulation

The BCI stimulation procedure was based on the Hex-o-Spell [19] implementation presented by Van Den Kerchove et al. [30]. During the experimental session, participants were comfortably seated in a chair or in their wheelchair in front of a table. Stimuli were presented on an Acer Predator Helios laptop with an 18" screen (Acer, Inc., Taiwan) placed at a 60 cm distance. Stimulation was performed using PsychoPy (version 2023.1.3) [33]. A Cedrus StimTracker (Cedrus Corp., CA, USA) ensured synchronization of stimuli with the recorded EEG.

The stimulation display shows 6 circular white targets with a diameter of 4.15° visual angle laid out on the corners of a hexagon with radius 12.28°. The task consists of counting the intensifications of a cued target, while fixating the gaze on a cued fixation cross. If the fixation cross was absent, the gaze could move freely during the counting task. Targets were intensified by scaling them to a larger size (5.50° of visual angle)

**Fig. 1:**
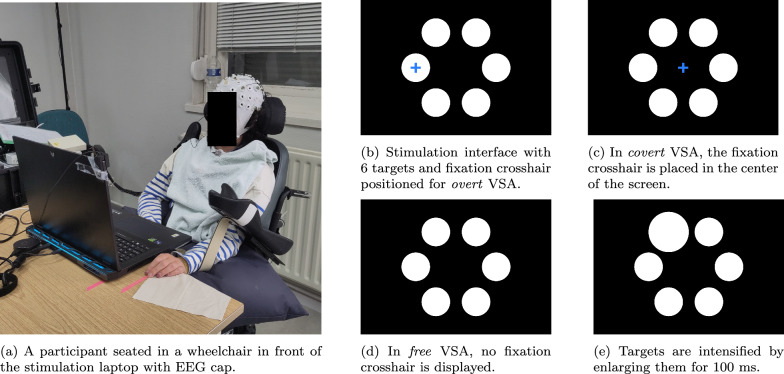
Stimulation and recording setup for the oddball BCI experiment with different VSA conditions

Using this system of an attention cue (the target to count), and a gaze cue (the fixation cross), three different VSA settings were explored as shown in Fig. 1. In the overt VSA setting, the participant was instructed to fixate on the cued target or to try this to the maximum extent of their capability or comfort. In the covert VSA setting, the participant was instructed to fixate on the center of the screen, to the extent of their ability. In addition to Van Den Kerchove et al. [30], an additional *free VSA* setting was introduced. Here, the participant was instructed to perform the task as they deemed most comfortable. This allowed us to investigate the user’s natural way of operating the BCI given their individual differences in vision and eye motor control. Participants were instructed to avoid head movements if they could do so voluntarily.

Stimulation was performed in blocks, one block for each cued target for each VSA condition, resulting in 6 * 3 = 18 blocks presented in randomized order. At the beginning of each block, the attention cue (the target to count) is indicated by a yellow cue. Next, the yellow arrow disappeared, and the gaze fixation cross was shown if applicable. This cross stayed visible throughout the block. The participant was asked to convey a simple signal if they were ready to start a block, and if they had memorized the attention cue and fixation cue, on which the experimenter could progress through the experiment at the pace of the participant.

After the cue, all 6 targets began intensifying in pseudorandom order. A target was intensified for a duration of 100 ms with an inter-stimulus interval (ISI) of 200 ms with added random jitter uniformly distributed between − 50 and 50 ms. Within a block, the intensification count of a target was randomly varied between 10 and 15 intensifications to keep the relevance of the counting attention task. Only the first 10 intensifications of each target were kept for analysis. After the stimulation phase of the block, the participant was asked for the number of intensifications counted to verify if they were performing the task correctly.

The experiment started with a practice block for each of the VSA conditions with instructions for each condition. During these practice blocks, the participant received oral feedback on their counting accuracy to ensure they understood and were able to perform the task. After block 6 and 12, the participant took a break with a minimum of 5 min.

### Data collection and preprocessing

During the experimental session, EEG and eye tracking signals were recorded. EEG was recorded at 1000 Hz using the Neuroscan Neuvo portable amplifier (Compumedics Neuroscan, Australia) connected to a second laptop for registration. The EEG headset used 18 active AgCl electrodes (EASYCAP GmbH, Germany) placed on a cap according to the international 10–20 layout. Using electrolyte gel, electrode impedances were reduced below 10 kΩ. Additionally, the electrooculogram (EOG) was recorded.

The EEG was band-pass filtered between 0.5 and 16 Hz. Bad channels were rejected using the RANSAC algorithm [34] and visual inspection. Next, the EEG was re-referenced to the average of mastoid electrodes TP9 and TP10, and independent component analysis (ICA) was performed to reject artifactual components based on correlation with the EOG or by visual inspection. For decoding, epochs were cut from − 0.1 to 0.9 s relative to stimulus onset, and no baseline correction was performed. Appendix D presents the ERPs obtained as the contrasts of the average target and non-target trials for each subject and VSA setting. These ERPs shown for visual interpretation originate from baseline-corrected epochs cut from − 0.2 to 1.0 s relative stimulus onset.

Eye tracking was performed using a Tobii X2-30 Compact (Tobii Technology AB, Sweden) portable eye tracker placed at the bottom of the laptop display. The registered data was cleaned by fusing left and right eye screen-based gaze coordinates into one channel for the horizontal and one for vertical gaze position. If both were present for a given sample, the fused channel was the mean of both values. If at a given sample either the left or the right eye was not detected for a given channel, the value of the other one was adopted. If both were missing, the gaze position remained unset at that time point, and no interpolation was performed.

### Data analysis

#### Range of comfortable eye motility

Research question 1 aims to explore the eye motor capabilities and usage during overt, covert and free visual BCI use. We clarify this by evaluating the eye gaze position of the participant with cued target gaze fixation (overt VSA), cued central fixation (covert VSA) and to assess the occurrence of these patterns when fixation is not cued (free VSA). After calibration, eye tracking follows the participant’s gaze throughout the BCI recording session when stimulation is active. The outcome of this analysis should be interpreted with care, as eye tracker calibration partially relies on intact eye motility. The participant’s position relative to the eye tracker might also frequently shift throughout the experimental session despite our best efforts, due to e.g. a regular need for aspiration of the tracheostoma of some participants.

#### BCI decoding

Research question 2 aims to establish a baseline BCI target selection decoding performance for the target user group. To this end, we perform off-line decoding of the cued target selections using the experimental protocol described in Sect. 2.3 and two state-of-the art decoding algorithms: block-Toeplitz linear discriminant analysis (tLDA) [35] and the Riemannian approach XDAWNCov+TS+LDA [36]. For decoding, cropping the epoch between 0 and 800 ms resulted in maximal accuracy.

Target selection accuracies are obtained using 6-fold cross-validation with folds corresponding to stimulation blocks. All target stimulus repetitions beyond 10 are dropped. We report both single-trial accuracy and accuracy averaged over specific numbers of repetitions of all 6 targets. In the single-trial case, a selection consists of 6 subsequent unique, allowing for more selections than 1 per block and more fine-grained accuracies. In the case of averaging over multiple trials, only one selection per block is made to allow for comparison over multiple repetitions as in realistic operation. The target and non-target epochs are separately averaged over these repetitions to enhance their signal-to-noise ratio.

Accuracies are reported with 95% confidence intervals, determined using 10,000 bootstrapping repetitions. To interpret the data, these confidence intervals were used to test hypotheses verifying which conditions or decoders were outperformed by others.

#### Gaze-independent BCI decoding

Research question 3 investigates the role that gaze-independent decoding algorithms could play in improving BCI decoding performance for individuals with SPGI. We repeat the decoding analysis above using a decoding algorithm specifically optimized for covert VSA decoding: Classifier-based Latency Estimation with Woody iterations (WCBLE) [30]. WCBLE is specifically designed for gaze-independent decoding by relying on latency jitter correction. 

WCBLE integrates a regular classifier with temporal alignment in an iterative scheme. First, the data is convolved with the trained regular classifier, in this case tLDA. From this convolution, classification score over time is extracted, and the latency with maximum classification score can be selected. These latencies can then be used to align the data to retrain the classifier for another iteration. To allow for proper latency estimation, a wider region of interest from -100 to 900 ms relative to stimulus onset was used.

#### Cross-condition decoding

Finally, research question 4 seeks an alternative approach to improve decoding performance. We propose the following approach: performance or comfort might be improved if calibration is performed using a given VSA setting, executed by relying maximally on the participant’s remaining gaze control. This way, informative properties depending on gaze fixation can be included in the decoder parameters, independent of gaze position during operation. This in turn might improve performance when different VSA conditions are performed during realistic BCI operation.

Off-line target selection accuracy was assessed as a metric of performance in a way similar to the BCI decoding analysis. However, the cross-validation scheme was altered to train the decoder on 5 blocks from one VSA condition and evaluated on a block from another condition. This way, all pairs of overt, covert and free VSA were evaluated.

## Results

### Included subjects and their visual capabilities

In total, 11 individuals were contacted. Of these, one person with Multiple Sclerosis (MS) was excluded based on criterion 3. A person recovering from traumatic brain injury (TBI) was excluded based on criteria 2 and 4, and one person recovering from stroke based on criterion 1. One further person recovering from a stroke was excluded due to technical difficulties during the experimental session. Ultimately, 7 participants were retained. Of these, one participant was diagnosed with bulbar-onset ALS, three with Friedreich’s Ataxia (FRDA) and three were recovering from LiS due to stroke. Table 1 lists the included participants and their diagnoses, each case is clinically described in appendix A. For these participants, the second visit of the study was carried out either in the hospital (A1), in their rehabilitation clinic (B1, B2, B4) or in their care home (C2, C3, C4).

**Table 1:** Included participants with their diagnosis and relevant communication capabilities.

ID	Diagnosis	Age	Sex	Speech	Primary communication method	W	KB
A1	Bulbar-onset ALS	58	M	Anarthric	Tablet with touchscreen	3	4
B1	FRDA	41	M	Dysarthric	Speech	3	3
B2	FRDA	43	F	Dysarthric	Speech	3	3
B4	FRDA	48	M	Dysarthric	speech	3	3
C2	Ischemic brainstem stroke	43	M	Anarthric	Eye movements	2	4
C3	Hemorrhagic brainstem stroke	43	F	Anarthric	Letterboard with finger pointing or touch	2	3
C4	Ischemic brainstem stroke	54	M	Anarthric	Letterboard with finger pointing or touch	2	3

W: classification according to Wolpaw et al. [31] used as inclusion criterion, KB: classification according to Küubler and Birbaumer [37] for further reference

Eye motor and visual differences assessments are reported in Table 2. All participants reported they experienced some degree of fatigue or discomfort when fixating. Participant A1 had the mildest impairment, only reporting fatigue when fixating for prolonged times. The participants with FRDA mostly exhibited eye tremors and impaired pursuit. Impairment of eye motor function was most prominent in participants C2, C3, and C4, those recovering from brain stem and cerebellar stroke.

**Table 2:** Vision and eye motor assessment as defined by Fried-Oken et al. [9] of participants included in this study.

	A1	B1	B2	B4	C2	C3	C4
Visual fixation	−	−	−	−	−	−	−
Eyelid function	+	+	+	+	+	−	−
Ocular motility	+	−	+	−	/	/	−
Binocular vision	+	+	+	+	−	/	/
Field of vision	+	+	+	+	+	/	−
Involuntary movement	+	/	/	−	−	/	+
Visual acuity	0.0	0.0	0.6	0.2	0.0	0.0	0.6

+ no impairment, − minor impairment, / major impairment. Visual acuity was assessed using the logMAR scale (0.0: normal vision, 0.05–0.5: minor visual impairment, 0.5–1.3: major visual impairment, > 1.3 blindness [38])

**Fig. 2:**
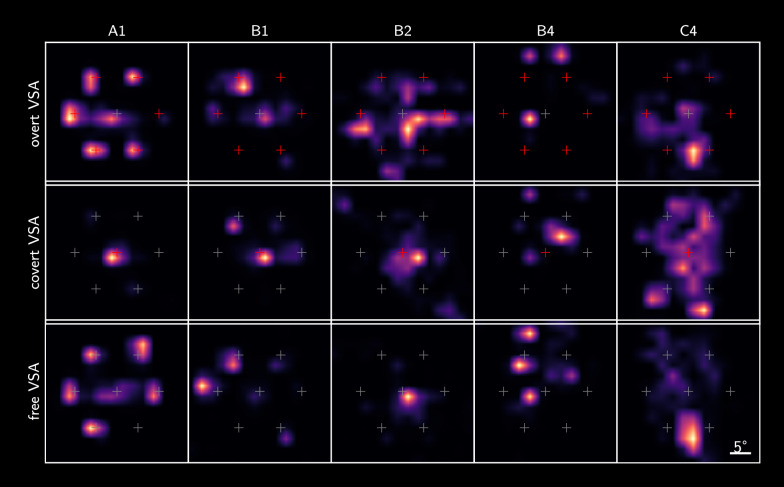
Distribution of the recorded gaze position during the experimental session in the three VSA conditions. Crosshairs represent stimulus positions, with positions cued during the given condition indicated in red. Subjects B2 and C4 preferred covert BCI operation, with B2 resting gaze near the middle of the screen, and C4 near the bottom. A high-resolution figure is available as Additional file 1

Figure 2 maps gaze position relative to the targets across conditions recorded during the BCI recording session. A1 had relatively intact gaze control and was able to correctly perform the cued overt and covert settings. When gaze was uncued, he fixated on the cued target. This was also mostly the case for B1, although eye tracking revealed that he chose not to perform central gaze fixation when cued in at least one of the stimulation blocks. We were unable to record his gaze near the bottom-left stimulus position, either due to eye tracker failure or because the participant was not comfortable fixating on this position. Eye tracker calibration did not succeed for subject B4, but given the transformation of gaze positions to the stimulus space, he was assumed to be overtly performing the free task.

B2 was able to perform overt VSA and central fixation to some extent, yet eye tracking shows a larger spread in gaze position compared to A1 and B1. In the free VSA condition, however, she preferred to attend stimuli covertly when the gaze was uncued. This was confirmed by the participant.

The overt and central gaze fixation settings were also not properly adapted to participant C4. In the free VSA condition, eye tracker results show that his gaze was usually near the bottom two targets, indicating some degree of covert or split VSA. Technical difficulties were encountered while recording gaze position with the Tobii X2-30 Compact for participants C2 and C3, since they both had one eye that was occluded respectively by the prism glass and the eyelid. Both participants reported they could not fixate on some of the stimuli.

As a complementary metric to the participants’ ability to perform the task, we questioned their experience of the experiment as reported in Table 3. Mood and tiredness were generally comparable before and after the task. Only participant B4 reported a mood decrease from 9 to 5. Contrary to our expectations, the experiment did not have a large impact on tiredness. Only participant A1 reported an increase from 6 to 7.

**Table 3:** Participant experience of the visual BCI task

Subject	A1	B1	B2	B4	C2	C3	C4
Mood before	6	6	7	9	10	10	10
Mood after	7	7	7	5	10	–	10
Tiredness before	6	5	7	6.5	3	10	9
Tiredness after	7	5	7	6.5	3	–	9

### BCI decoding performance

**Fig. 3:**
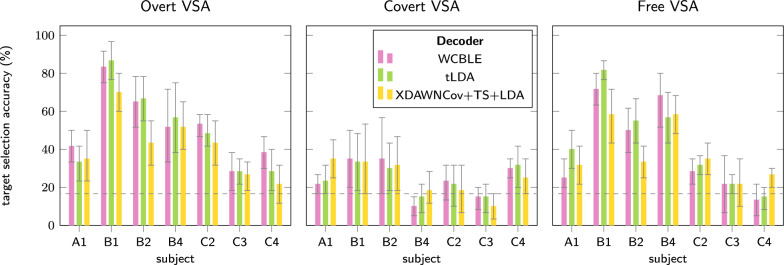
Comparison of single-trial target selection accuracies between the evaluated decoders. Decoders were tested in the three different VSA settings. Dashed lines indicate chance level accuracy (1/6). No single decoder yields substantially better performance than the others

Figure 3 shows cross-validated single-trial target selection accuracy for the evaluated VSA settings for the different decoders. A full table of results is available as Additional file 2. Accuracy results do not reveal a clear trend in decoder performance per condition. In the covert VSA setting with cued central gaze fixation, accuracy was lower overall. Target selection accuracy for this task was around chance level (1/6) for participants B4, C2 and C3. WCBLE did not improve over the accuracy of tLDA in the free VSA setting, but XDAWNCov+TS+LDA accuracy was slightly lower here, though not significantly. Therefore, we select the tLDA decoder for further analyses as the most suited decoder because of its simplicity.

**Fig. 4:**
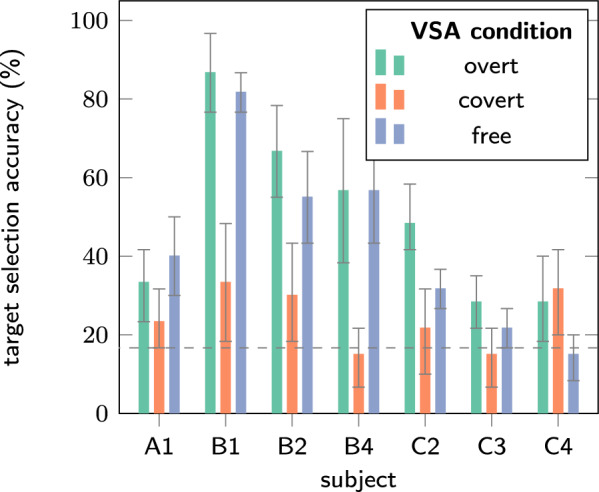
Decoding accuracy compared across VSA conditions using the tLDA decoding algorithm. Covert VSA generally performs poorly, but free and overt VSA are often on par, except for participant C2, who performed best with overt attention.

More interestingly, we noticed that accuracies of the decoders in free VSA were close to those in the overt VSA as displayed in Fig. 4. A substantial decrease in accuracy from the overt setting to the free setting was observed for subjects C2, C3 and C4 who had the most severe eye motor impairment. For B2, who relied on covert VSA during the uncued free VSA according to gaze tracking setting, the decrease in accuracy was also present.

Finally, we report target selection accuracy obtained by averaging multiple trials, a strategy often applied in BCIs. This gives a more realistic insight into decoding performance. Full results are reported in appendix E. Table 4 reports the number of repetitions needed to reach 80% accuracy, a commonly accepted threshold for effective BCI decoding [20]. In covert VSA, only participants B1 and C4 managed to reach acceptable performance, with a high amount of repetitions, 10 and 9 respectively. In both free and overt VSA, most participants managed to achieve acceptable performance after some repetitions.

**Table 4:** Number of repetitions needed to reach 80% accuracy with the tLDA decoder.

Subject VSA condition	A1	B1	B2	B4	C2	C3	C4
Covert	–	10	–	–	–	–	9
Free	6	1	3	2	9	–	–
Overt	9	1	3	2	3	7	–

### Cross-condition decoder training and evaluation

**Fig. 5:**
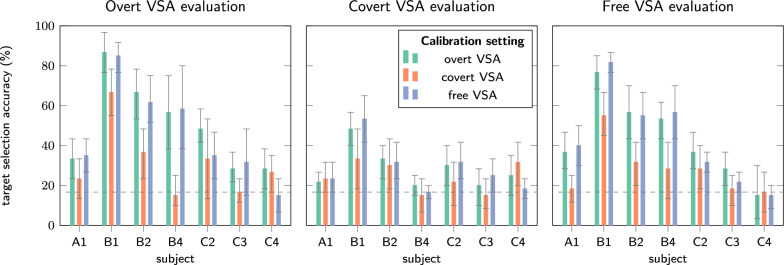
Single-trial target selection accuracy when calibrating the tLDA decoder in a given VSA setting, and evaluating it in another. Whiskers indicate confidence intervals. For participants A1, B2, and C3, decoding accuracy in the covert VSA setting with central gaze fixation improved when calibrating with overt gaze fixation.

Figure 5 shows that for participants B1 and C2 covert VSA decoding improved when training with overt VSA. Note that, according to eye tracking data, participant B1, B4, and C4 did not always perform cued central gaze fixation in the covert VSA setting, which might have affected the results. A full table of results is available as Additional file 3.

## Discussion

Due to the heterogeneous nature of the participants’ conditions and the limited number of participants, it is difficult to draw general conclusions. This study should therefore be seen as a collection of case studies, highlighting different obstacles encountered in developing gaze-independent visual oddball BCIs for individuals with SPGI. Nevertheless, we highlight some aspects that might be of interest for the further development of this class of BCIs.

This study investigated the feasibility of gaze-independent visual oddball BCIs for users with speech physical and gaze impairment (SPGI). To the authors’ knowledge, there are few studies that recruited realistic cases with a variety of SPGI etiologies, and thoroughly assess their use of visual, ERP-based BCIs with a spatial layout. We establish insights into their performance, needs and behavior, without assuming specific operation or eye fixation conditions a priori. We go further by relating their specific clinical, visual and eye motor context and gaze tracking results to their performance. While other studies merely point out the drawbacks or introduce alternative paradigms with lower ITR, we investigate gaze-independent decoders and calibration schemes for classical, spatially-organized interfaces that could provide solutions relevant to the target population. While we did not confirm earlier results on WCBLE as performant gaze-independent decoding algorithm, the cross-condition calibration scheme with overt VSA calibration improves performance.

### Gaze-independent operation and decoding

True *gaze-independent* visual BCIs should not rely on gaze fixation. Hence, our analysis centers around the free VSA condition. Eye tracking results presented in Sect. 3.1 confirm our assumption that voluntary covert VSA can occur in SPGI. We found mild evidence for the results presented by Van Den Kerchove [30], which state that the WCBLE decoder can improve performance in covert VSA with central gaze fixation. This holds for two participants, A1 and C4.

Contrary to our assumptions, we have shown that accounting for latency jitter does not necessarily improve covert VSA when gaze fixation is not cued. One possible explanation is that actively performing central gaze fixation increases task load. This, in turn, can reduce overall performance, even though the participant might have otherwise performed covert VSA, but would not be occupied with maintaining strict central gaze fixation. This extra task demand raises questions about the relevance of this condition. Furthermore, cued central gaze fixation combined with counting flashing stimuli in the visual periphery is an explicit example of a dual task. Dual tasks have been shown to increase latency jitter of the P3 ERP component [28, 30, 39], which is what WCBLE accounts for. Hence, increased P3 jitter might be more related to maintaining central gaze fixation than to the actual covert VSA aspect.

The seemingly stable accuracy across overt and free VSA could be misinterpreted as an indication that the Hex-o-Spell BCI already works well for individuals with SPGI, and no optimization is needed. However, we assume that overt VSA accuracy was also decreased in some subjects or for some blocks if the participant was not able to comfortably perform the task. Nevertheless, the large difference between the free VSA setting and the covert VSA setting with central gaze fixation raises questions about the applicability of solutions developed with central fixation in mind.

Covert VSA with cued, central gaze fixation resulted in reduced decoding accuracy, likely due to an increased task load. Accuracy in free VSA, where gaze position is uncued, can perform on par with explicitly cued overt VSA for some participants. Most individuals facing gaze impairment will likely retain some capability to direct their gaze in visual BCI operation, which can drastically boost accuracy. Participant B2 exemplifies this: her free VSA decoding accuracy is above that in covert VSA, and eye tracking showed that she relied mostly on overt VSA when cued to do so, and mostly on covert VSA when gaze was uncued.

The importance of relying on residual motor capability is also supported by our results on cross-condition decoder training and evaluation presented in Sect. 3.3. Fixating targets during the decoder training phase can improve decoding accuracy in some settings. This is likely due to the increased P3 component amplitude in overt VSA, which improves the discriminative power of a classifier trained on this data. Cueing this overt gaze fixation only during the decoder training phase leaves the user free to operate in the manner that is most comfortable in the operation phase. The presence of early, visual ERP components in the training data can then also contribute to performance in those cases where the participant was not able to perform covert VSA with central gaze fixation.

### Clinical implications

The population of individuals with SPGI is relatively scarce, yet is regularly confronted with major challenges. This study does not focus on individuals living in a vegetative or minimally conscious state, or with complete LiS. Further development of the proposed technology might not necessarily be helpful for them. Aside from practical considerations, our results show that pure covert VSA performs subpar, even in individuals with substantial retention of eye motor control. Another reason to shift the focus away from complete LiS is the *extinction of thought* phenomenon [37]. This condition refers to a decrease in voluntary goal-directed cognitive activity, which calls into question the users’ intent to engage in communication at all.

Instead, the focus lies on individuals who actively use or intend to use AAC, but experience specific barriers with existing methods. These users demonstrably have the intent and partial capability to communicate their thoughts, opinions and desires to their surroundings, including but not limited to clinicians, caregivers and their social network. While they experience a need for efficient and effective communication, this need is often unmet due to the lack of suited AAC technology. Hence, finding a way to fill in this gap is a major issue in care for individuals with SPGI. Some of these people who use or intend to use AAC might benefit from visual BCIs for at-home use or in a clinical setting. While the proposed communication protocol is a proof of concept with a limited degree of freedom, it is a step towards applications like textual communication and environment or home automation control that inherits the relatively high information transfer rate of visual BCIs.

When specifically considering individuals with eye motor impairment, it is important to thoroughly assess their needs, capabilities and eye motor skills on the one hand, and, on the other hand, identify obstacles for practical deployment. Our results indicate that some of the evaluated participants naturally rely in part on gaze control. It is important to consider this in any practical AAC solution, which should maximally rely on the capabilities of the user. This can also be done in other ways, e.g. by integrating residual muscle control, eye tracking or EOG. In general, gaze-independent BCIs show promise for users with different degrees of eye motor control, but the exact solution should be specifically adapted to each individual user separately and properly integrated with a suited AAC solution. It is important to also consider other strategies known in the BCI field than those proposed here, focusing on properly choosing the paradigm, the decoder and the calibration strategy.

The result on mood and tiredness, combined with our interaction with the participants revealed that the technology and its potential applications were generally well received by both the participants themselves and their social environment. It is clear that visual attention tasks can be taxing if performed for extended periods of time, especially in the presence of SPGI. Yet, participants subjectively indicated that this was outweighed by the potential to communicate in a more automated and autonomous way compared to their current AAC solutions, which more often required the help of a trained caregiver. Nevertheless usability and comfort remain critical considerations for real-world applications. In summary, the provided results offer important insights into the application of AAC technology for individuals with severe (eye) motor impairments, and underscore the need for further research into adaptive communication strategies and evaluating user satisfaction.

### Limitations and recommendations

Despite the insights gained on gaze-independent BCI approaches, certain limitations should be noted. First and foremost, this study works with a limited sample size, which does not cover the full SPGI spectrum and specific symptoms and eye movement control differences that might occur. Individuals with FRDA met the inclusion criteria, but they are usually not considered a typical interest groups for BCI-AAC, partly due to the rarity of the disease, and partly due to the characteristics of its progression and symptoms. It would be particularly interesting to verify these results with individuals with complete LiS, who retain no eye movement capability at all.

Another limiting factor is the difficulty experienced in correctly interpreting eye tracker results in the presence of gaze impairment. If eye tracking is possible at all, it is not guaranteed that the participant or end user is able to successfully perform the calibration procedure. Further experiments should be carried out with a stationary eye tracker with more advanced capabilities, although systems using a head fixator or chin rest should be avoided. This is not practical when working with individuals who are usually seated in a wheelchair, who may have undergone a tracheostomy or may have spasticity in certain limbs or the chest. Given different, more suitable gaze tracking hardware, adapted to the participant’s conditions, analyses could be performed with a finer granularity by assessing the gaze condition on a per-epoch basis.

In this study, user comfort in the different conditions was measured rather subjectively and generally. Additionally, we assumed that participants operated most comfortably in the free VSA condition. Even though participants reported that they could comfortably operate the system, this must be confirmed with more quantitative assessments. To properly contextualize performance results, they should be coupled with metrics evaluating the full range of the user’s requirements, with measures of usability, comfort and perceived effort. Feedback should be collected with a more fine-grained method to allow for comparison of conditions within the experiment. More structured evaluation methods include the NASA Task Load Index [40], the QUEST 2.0 AAC satisfaction questionnaire [41] and the feedback received through Assistive Technology Device Predisposition Assessment [42]. Decoding accuracy might, after all, be traded for user comfort.

The perception of this type of BCI by the user might also be influenced by performing the experiment in an on-line manner, providing immediate feedback after selection and thus closing the loop. Follow-up work should investigate this in a longitudinal study following realistic communication and at-home on-line use of visual BCI AAC devices.

Eye motor function could also have been assessed more quantitatively and objectively [9], using, e.g., the Revised Coma Recovery Scale [43], the NSUCO oculomotor exam [44], or the eye-tracking based, computerized NeuroEye test [45], in order to relate them to the decoding or satisfaction results.

To optimize BCI user experience, research must extend beyond offline classification performance and address the full scope of user interaction during online use. Offline evaluation, while convenient, fails to capture critical factors such as user engagement, learning effects, and genuine satisfaction. A user-centered design (UCD) framework offers a structured approach to evaluate effectiveness, efficiency, and satisfaction in realistic settings [46–49]. This includes transitioning from abstract stimuli to meaningful interfaces providing immediate feedback, and evaluating performance metrics in real-time. Satisfaction, a subjective yet vital component, should be assessed through questionnaires after realistic online use [48]. Decoder development, interface design, and paradigm selection should be co-optimized, as improvements in decoding alone may be insufficient if the paradigm does not align with the user’s abilities and preferences. Ultimately, the goal is to design a usable system accounting for specific individual differences, where longitudinal studies with target user cases or groups are required to evaluate system performance comprehensively.

Implementing such a user-centered approach requires early and active involvement of individuals with SPI throughout the research process [46]. Instead of a traditional bottom-up approach—starting with healthy controls and offline analysis—similar future projects should begin directly with online experiments in the target population, identifying challenges and iteratively optimizing the system [9]. This requires long-term collaboration with patient centers to ensure an involved team of clinicians, occupational and speech therapists, and other caregivers, next to further arrangements like ethical approval, and access to suitable infrastructure from the onset. Maintaining a functional, in-house, online BCI-AAC system facilitates iterative development and realistic performance estimation, ensuring that experimental settings reflect practical use. While this top-down approach may not suit novel BCI paradigms in early development, it is appropriate for mature paradigms such as visual oddball BCIs [50, 51], where the main challenge lies in usability and disseminating the technology to potential users.

## Conclusion

This study explored the usability of electroencephalography (EEG)-based gaze-independent visual BCIs in individuals with speech, physical and gaze impairment (SPGI), focusing on the impact of eye motor impairments on performance. Our results demonstrate that a visual brain–computer interface (BCI) with gaze-independent operation is feasible for some of these individuals. They might either achieve sufficient decoding accuracy despite their eye motor impairment, or performance can be enhanced by careful, individual adaptation of the decoding strategy. The free visuospatial attention (VSA) condition yielded decoding accuracy comparable to overt VSA in some participants, suggesting that users may naturally integrate remaining gaze control. Decoding algorithms specifically designed for covert VSA have limited impact, but calibrating in the overt VSA condition improved performance in the subsequent, more comfortable free VSA operation. This highlights the potential benefits of leveraging remaining eye motor capabilities during decoder training.

While these findings are promising, future work should include larger participant groups, refine stimulation paradigms, and incorporate real-time, user-centric assessments of comfort and usability to actively develop usable solutions. Ultimately, optimizing gaze-independent BCI designs could enhance communication options for users experiencing severe motor and speech impairments, particularly those experiencing difficulties controlling conventional eye tracking technologies.

## Supplementary Information

**Additional file 1.** Heatmap of gaze location per VSA condition. *Description*: High-resolution heatmap of the distribution of recorded gaze position during the experimental session in the three VSA conditions. Crosshairs represent stimulus positions, with positions cued during the given condition indicated in red.

**Additional file 2.**. Decoding performance per participant. *Description*: Full table of decoding target selection accuracies per participant, decoder and VSA condition

**Additional file 3.**. Cross-condition decoding performance per participant. *Description*: Full table of cross-validated, cross-condition decoding target selection accuracies per participant and decoder.

## Data Availability

The datasets generated and/or analysed during the current study are not publicly available due to the policies in agreement with the ethical approval to protect sensitive personal information, but can be obtained from the corresponding author upon reasonable request.

## Appendix A: Clinical evaluation of participants

**A1** *Diagnosis:* 58 y.o., originally left-handed male diagnosed with bulbar-onset Amyotrophic Lateral Sclerosis (ALS) 2 years prior at age 56. Recently, symptoms started progressing more on the right side and the participant lost voluntary control the left limbs and partially of the right limbs, but right arm and finger movement were possible, albeit with effort. *Presentation:* A1 was lying reclined in a hospital bed, the experimental setup was positioned on a movable folding table. *Communication:* A1 was anarthric, and communicated using vocal grunts and a text-to-speech AAC tablet operated with the right hand. *Eye movements:* He reported fatigue linked to his disease progression when visually fixating for prolonged times, but otherwise reported no eye movement abnormalities or visual defects.

**B1** *Diagnosis:* 41 y.o., originally left-handed male diagnosed with Friedreich’s Ataxia (FRDA) 29 years prior, at age 12. Symptoms resulted in slowed, effortful body and limb movement and affected fine motor control, and most severely affected the left side which made him effectively right handed. *Presentation:* B1 was seated in an electric wheelchair, which was positioned in front of a table with the experimental setup. *Communication:* Verbal communication was possible, but dysarthria resulted in slowed, slurred speech. He was able to effectively control a PC and a smartphone, but writing was difficult. *Eye movements:* B1 reported impaired visual pursuit of moving targets and fatigue and discomfort fixating. Indeed, we noticed effort when making eye movements, which resulted in large muscular artifacts registered in the EEG and electrooculogram (EOG) Additionally, he reported minor nystagmus which we also noticed in the horizontal EOG.

**B2** *Diagnosis:* 43 y.o., right-handed female diagnosed with FRDA symptom-free at age 9, with symptom onset at age 12, 31 years prior to the experiment. Symptoms resulted in slowed, effortful body and limb movement and affected fine motor control. *Communication:* Dysarthric, characterized by large effort necessary to speak. Hand signaling was also often used, but also required effort. *Eye movements:* B2 was affected by especially severe horizontal eye oscillations with onset 10 years prior to the experiment. Fixating was also fatiguing and uncomfortable, especially when not wearing glasses or lenses.

**B4** *Diagnosis:* 48 y.o., right-handed male diagnosed with FRDA 30 years prior at age 18. *Presentation:* B4 was a wheelchair user but could stand up and move into position for the experiment without support. *Communication:* Speech was relatively fluent, only slightly slurred, leading to mild dysarthria. *Eye movements:* Pursuit and fixation were impaired, as characterized by self-reported fatigue and discomfort. Nystagmus. B4 wore no corrective glasses lenses.

**C2** *Diagnosis:* 43 y.o., originally right-handed male diagnosed with Locked-in Syndrome (LiS) due to ischemic brain stem stroke 6 years prior, at age 37. He underwent a tracheostomy. *Presentation:* C2 was seated in an electric wheelchair controlled by a caregiver, and his head was fixated in a headrest. This headrest contacted the EEG cap occipitally and centrotemporally, which interfered slightly with cap and electrode positioning and optimization. Additionally, heavy movement artifacts were noticed in the EEG due to the combination of breathing, aspiration, the headrest and maintaining correct positioning for the experiment. The EEG cap was held in place using a chest strap. *Communication:* C2 had complete anarthria and had no preserved facial movement. He communicated with caregivers by responding to prompted letters and words with upwards eye movement signals. Additionally, he sometimes made use of an emergency button which he could control with his left hand, the only extremity in which he had some residual control capability. *Eye movements:* Right eye: preserved vertical movement, paralysis of laterality to the right and to the left, which resulted in the inability to perform horizontal movements. Left eye: preserved vertical movement, adduction possible, abduction deficit. Deviation in the left eye resulting in diplopia, corrected by a prism lense. Self-reported tremors and fixation fatigue and discomfort. Eye tracker calibration did not succeed.

**C3** *Diagnosis:* 43 y.o., right-handed female diagnosed with hemorrhagic stroke 4 years prior, at age 39. She was tetraplegic and underwent a tracheostomy. *Presentation:* C3 was seated in an electric wheelchair *Communication:* C3 was completely anarthric, but had sufficient residual motor control to indicate letters and symbols on a letterboard by pointing with movements of the right index finger and hand. *Eye movements:* Right eye: complete ptosis. When corrected with lenses, downward movement was preserved and incyclotorsion was possible through preserved cranial nerve IV. Ophthalmoplegia in other directions. Left eye: limited adduction, complete verticality, horizontal nystagmus. Intermittend nystagmus. Self-reported involuntary saccades and fixation fatigue or discomfort.

**C4** *Diagnosis:* 54 y.o., originally right-handed male diagnosed with ischemic brainstem stroke 11 years prior, at age 53. He is tetraplegic and underwent a tracheostomy. Symptoms most pronounced on left side. *Presentation:* The tracheostomy resulted in frequent aspiration which interfered with the experiment and recording. This and other factors related to the condition and positioning in the wheelchair resulted in large and frequent muscular and movement artifacts in the EEG. Head bobbing when concentrating on the experimental task. *Communication:* C4 had sufficient residual motor control to indicate letters and symbols on a letterboard by pointing with movements of the right index finger and hand. *Eye movement:* A corneal abcess in the left eye affected its motility and resulted in deviation. The left eye had poor vision (only able to distinguish shapes) and could not be closed. The right eye was not affected. The deviation resulted in fatigue and discomfort fixating. Hypermetropia. The participant wore corrective lenses. Eye tracker calibration did not succeed.

## Appendix B: Questionnaire for basic clinical evaluation

In addition to the information provided by the paramedical team, the supervising clinician and the information obtained through observation by the experimenters, the following information was gathered on a structured basis:

1. Age
2. Handedness
3. Sex
4. Neuromuscular diagnosis
5. Time of neuromuscular diagnosis if applicable
6. Stage of the neuromuscular disease if applicable
7. Ophtalmological diagnosis
8. Time of ophtalmolocical diagnosis if applicable

## Appendix C: Questionnaire for self-reported eye motor assessment

In addition to the visual acuity test and the information obtained from the medical file or the oculomotor assessment provided by the clinician, the following questions were asked to poll for self-reported eye movement and vision difficulties.

1. Do you experience any fatigue or discomfort when moving the eyes?
2. Do you experience any fatigue or discomfort when fixating the gaze?
3. Do you experience any involuntary eye movements, including tremors jitters or larger movements or drifts?
4. Do you experience discomfort or the inability to perform eye movements in specific directions?
5. Are there any parts of the field of view that you cannot or cannot comfortably view, redirect the eyes to or gaze at?
6. Do you experience any other issues with vision?
7. Do you experience any other issues with eye movement?

## Appendix E: Accuracy over repetitions

See Table 5.

**Table 5:** Accuracy of the block-Toeplitz linear discriminant analysis (tLDA) decoder when averaging over multiple repetitions

Subject	Number of repetitions VSA condition	1	2	3	4	5	6	7	8	9	10
A1	Covert	0.17	0.17	0.17	0.33	0.33	0.33	0.17	0.33	0.50	0.33
	Free	0.33	0.50	0.50	0.67	0.50	0.83	0.83	0.83	1.00	1.00
	Overt	0.33	0.33	0.33	0.50	0.67	0.50	0.33	0.67	0.83	0.83
B1	Covert	0.50	0.50	0.33	0.33	0.33	0.50	0.50	0.50	0.67	0.83
	Free	0.83	0.83	0.83	1.00	1.00	1.00	1.00	1.00	1.00	1.00
	Overt	1.00	1.00	1.00	1.00	1.00	1.00	1.00	1.00	1.00	1.00
B2	Covert	0.50	0.33	0.50	0.33	0.33	0.17	0.33	0.33	0.33	0.33
	Free	0.50	0.67	0.83	0.67	0.67	0.67	0.83	0.83	0.83	1.00
	Overt	0.33	0.67	1.00	1.00	1.00	1.00	1.00	1.00	1.00	1.00
B4	Covert	0.17	0.00	0.17	0.00	0.00	0.00	0.00	0.00	0.00	0.00
	Free	0.67	0.83	1.00	0.83	1.00	1.00	1.00	1.00	1.00	1.00
	Overt	0.67	1.00	1.00	1.00	1.00	1.00	1.00	1.00	1.00	1.00
C2	Covert	0.33	0.33	0.17	0.17	0.33	0.50	0.50	0.50	0.50	0.50
	Free	0.33	0.67	0.67	0.50	0.50	0.67	0.67	0.67	0.83	0.83
	Overt	0.67	0.67	1.00	1.00	1.00	1.00	1.00	1.00	1.00	1.00
C3	Covert	0.17	0.17	0.17	0.17	0.17	0.00	0.17	0.17	0.33	0.17
	Free	0.33	0.00	0.33	0.50	0.33	0.33	0.00	0.33	0.17	0.50
	Overt	0.50	0.17	0.33	0.50	0.50	0.67	0.83	0.67	0.83	0.67
C4	Covert	0.33	0.17	0.17	0.33	0.33	0.33	0.33	0.50	0.83	0.83
	Free	0.00	0.00	0.00	0.00	0.00	0.00	0.00	0.00	0.00	0.00
	Overt	0.17	0.50	0.67	0.67	0.67	0.67	0.67	0.67	0.67	0.67

## Abbreviations

AAC: Augmentative and alternative communication
ALS: Amyotrophic lateral sclerosis
BCI: Brain–computer interface
EEG: Electroencephalography
EOG: Electrooculogram
ERP: Event-related potential
FRDA: Friedreich’s Ataxia
ICA: Independent component analysis
ISI: Inter-stimulus interval
ITR: Information transfer rate
LiS: Locked-in syndrome
MS: Multiple sclerosis
RSVP: Rapid serial visual presentation
SPGI: Speech, physical and gaze impairment
SPI: Speech and physical impairment
SSVEP: Steady-state visually evoked potential
TBI: Traumatic brain injury
tLDA: Block-Toeplitz linear discriminant analysis
UCD: User-centered design
VSA: Visuospatial attention
WCBLE: Classifier-based latency estimation with Woody iterations
